# Tumor Necrosis Factor dynamically regulates the mRNA stabilome in rheumatoid arthritis fibroblast-like synoviocytes

**DOI:** 10.1371/journal.pone.0179762

**Published:** 2017-07-14

**Authors:** Konstantinos Loupasakis, David Kuo, Upneet K. Sokhi, Christopher Sohn, Bethany Syracuse, Eugenia G. Giannopoulou, Sung Ho Park, Hyelim Kang, Gunnar Rätsch, Lionel B. Ivashkiv, George D. Kalliolias

**Affiliations:** 1 Arthritis and Tissue Degeneration Program and David Z. Rosensweig Genomics Research Center, Hospital for Special Surgery, New York, United States of America; 2 Graduate Program in Physiology, Biophysics and Systems Biology, Weill Cornell Graduate School of Medical Sciences, New York, United States of America; 3 Computational Biology Program, Sloan Kettering Institute, New York, United States of America; 4 Biological Sciences Department, New York City College of Technology, City University of New York, Brooklyn, United States of America; 5 Department of Computer Science, ETH Zürich, Zürich, Switzerland; University of Toronto, CANADA

## Abstract

During rheumatoid arthritis (RA), Tumor Necrosis Factor (TNF) activates fibroblast-like synoviocytes (FLS) inducing in a temporal order a constellation of genes, which perpetuate synovial inflammation. Although the molecular mechanisms regulating TNF-induced transcription are well characterized, little is known about the impact of mRNA stability on gene expression and the impact of TNF on decay rates of mRNA transcripts in FLS. To address these issues we performed RNA sequencing and genome-wide analysis of the mRNA stabilome in RA FLS. We found that TNF induces a biphasic gene expression program: initially, the inducible transcriptome consists primarily of unstable transcripts but progressively switches and becomes dominated by very stable transcripts. This temporal switch is due to: a) TNF-induced prolonged stabilization of previously unstable transcripts that enables progressive transcript accumulation over days and b) sustained expression and late induction of very stable transcripts. TNF-induced mRNA stabilization in RA FLS occurs during the late phase of TNF response, is MAPK-dependent, and involves several genes with pathogenic potential such as *IL6*, *CXCL1*, *CXCL3*, *CXCL8/IL8*, *CCL2*, and *PTGS2*. These results provide the first insights into genome-wide regulation of mRNA stability in RA FLS and highlight the potential contribution of dynamic regulation of the mRNA stabilome by TNF to chronic synovitis.

## Introduction

Chronic sterile synovial inflammation and *pannus* are the hallmarks of rheumatoid arthritis (RA) [1]. *Pannus* is the expanded and inflamed synovial lining that invades adjacent bone and cartilage [2]. It is hypercellular, consisting of activated macrophages (Mφ) that secrete Tumor Necrosis Factor (TNF), and numerous fibroblast-like synoviocytes (FLS) that respond to paracrine TNF, establishing a Mφ-TNF-FLS axis. The effectiveness of biologics targeting TNF in RA suggests that the Mφ-TNF-FLS axis is at the epicenter of disease pathogenesis [3]. During the long-standing course of RA synovitis, chronic exposure to TNF and other inflammatory factors transforms FLS into synovial factories secreting a constellation of arthritogenic mediators [4,5]. These mediators induce synovial recruitment, retention, activation and prolonged survival of immune cells, and promote osteoclastogenesis, cartilage degradation as well as synovial neoangiogenesis [6].

In target cells, TNF triggers a series of molecular events that unfold in a stereotypic temporal order including an immediate-early phase followed by a later phase [3,7–9]. The acute molecular cascades triggered during the early phase have been extensively studied in many cell types [3]. In contrast, the late molecular events that are induced by TNF are largely unknown. In previous studies, we have shown that one single pulse with TNF triggers in RA FLS prolonged activation of NF-κB, sustained chromatin accessibility in the promoters of *IL6* and *CXCL10*, non-terminating transcription of *IL6*, and continuous expression of cytokines, chemokines and tissue destructive enzymes [10,11]. Within the chronically inflamed RA synovium, FLS are exposed to long-term inflammatory stimulation and their gene expression transitions from an early to a late program that shapes aspects of their aggressive phenotype.

In this study, we further investigate the molecular events induced in RA FLS during their transition from early to the late phases of TNF response, and elucidate the dynamics of regulation of gene expression. As our previous studies explored upstream signaling, chromatin modifications, and gene transcription [10,11], here we investigated the role of mRNA stability in the TNF response in FLS. Intrinsic differential stability of mRNA transcripts is well established to play an important role in cytokine-induced gene expression [8]; however, very little is known about the effects of inflammatory cytokines such as TNF on long term stability of specific transcripts during the late phase response. Strikingly, RNA sequencing (RNA-seq) and genome-wide analysis revealed that TNF induces prolonged stabilization of a subset of inflammatory gene transcripts. Dynamic regulation of the mRNA stabilome by TNF exhibited a temporal switch from an early phase dominated by unstable inducible transcripts to a late phase characterized by accumulation of stabilized and stable transcripts with pathogenic potential. To our knowledge, this is the first genome-wide study implicating mRNA stability in the sustained inflammatory response of primary RA FLS.

## Materials and methods

### Patients

Synovial tissues were obtained from RA patients who underwent, as part of standard medical care, total knee replacement or elbow synovectomy, using a protocol approved by the Hospital for Special Surgery Institutional Review Board that adheres to NIH guidelines and regulations. De-identified specimens that would otherwise have been discarded were used in this study and were obtained under a waiver of consent. The diagnosis of RA was based on the 1987 American College of Rheumatology criteria [12]. The results described in the manuscript have been verified in several independent experiments with cells derived from more than 10 different RA patients.

### Cell purification

Synovial tissue fragments were incubated with liberase. Cells were allowed to adhere to tissue culture dishes and passaged every 3–5 days. 4–5 passages yielded a relatively homogeneous population of FLS.

### Cell culture

FLS were cultured in alpha-MEM (plus 10% FBS, 1% Glutamine, and 1% Penicillin/Streptomycin). The following reagents were used as indicated: TNF (10 ng/ml; Pepro Tech), Actinomycin D (10 μg/ml; SIGMA), Triptolide (1 μM; SIGMA), Flavopiridol (0.5 μM; SIGMA), SB202190 (10 μM; Calbiochem), JNK Inhibitor II SP600125 (10 μM; Calbiochem), U0126 (10 μM; Calbiochem). Dimethyl sulphoxide (DMSO) (Sigma-Aldrich) was used as a vehicle control.

### Real-time quantitative RT-PCR (qPCR)

RNA was extracted from 0.5x10^6^ FLS using RNeasy mini kit (Qiagen) and 1μg was reverse transcribed using a First Strand cDNA synthesis kit (Fermentas). qPCR was performed using SYBR Green supermix following the manufacturer's protocols, and triplicate reactions were run for each sample. The human oligonucleotide primers used were:

GAPDH: 5-ATCAAGAAGGTGGTGAAGCA-3 and 5-GTCGCTGTTGAAGTCAGAGGA-3;
IL-6: 5-TAATGGGCATTCCTTCTTCT-3 and 5-TGTCCTAACGCTCATACTTTT-3
IL-6 Primary Transcript (PT): 5-GGACAACTCAGGGATGCAAT-3 and 5-GCAGAAGAGAGCCAACCAAC-3
IL8: 5-TTTTGCCAAGGAGTGCTAAAGA-3 and 5-AACCCTCTGCACCCAGTTTTC-3
CCL2: 5-AGCAGCAAGTGTCCCAAAGA-3 and 5- GTGTCTGGGGAAAGCTAGGG
CCL5: 5-GAGGCTTCCCCTCACTATCC-3 and 5-CTCAAGTGATCCACCCACCT-3
CCL5 PT: 5-TCCTGAGACCCTGAGACAGC-3 and 5- TGTGCCAAAATCAGCACAAT-3
CXCL1: 5-AGTCATAGCCACACTCAAGAATGG-3 and 5-GATGCAGGATTGAGGCAAGC-3
CXCL3: 5-TCCCCCATGGTTCAGAAAATC-3 and 5-GGTGCTCCCCTTGTTCAGTATCT-3
PTGS2: 5-GTGGCTGAACAAATTAACGAA-3 and 5-AGCCTGAATGTGCCATAAGA-3
MMP3:5-TCTCCTGCCTGTGCTGTG-3 and 5-CAGATTCACGCTCAAGTTCC
MMP3 PT: 5-ACAGGTTGATTCCTGGGTCA-3 and 5- CCCATATATGCCTGCTGTCC-3

### RNA-sequencing protocol and alignment

Total RNA was extracted from synovial fibroblasts derived from two different RA patients using the RNeasy mini kit (Qiagen). RNA-sequencing libraries were generated from two patient donor FLS using Tru-Seq kits (Illumina) for poly-A selected transcripts. Single end sequencing was performed (Illumina HiSeq 2500, 50 bp read lengths). Reads were aligned to the human reference hg19 using TopHat2 [13]. Quality control of aligned reads was performed using FastQC [14]. Reads per kilobase transcriptome per million mapped reads (RPKM) quantification was performed using the RefSeq annotation and CuffDiff2 [15]. Gene count quantification was performed using HTSeq [16]. The database of Genotypes and Phenotypes (dbGaP) was used for data deposition (dbGaP accession number: phs001371.v1.p1).

### Differential gene expression and pathway analysis

Count tables generated from HTSeq were processed and filtered based on expression in TNF treated conditions (>100 counts per gene). Differential testing was performed between TNF treated libraries in 1, 3, 24, 72 h, and the control libraries using DESeq2 [17]. DESeq2 significance thresholds were set to adjusted p-value<0.1. Significant differentially expressed, differentially induced or differentially stabilized gene lists were generated and used as input for pathway analysis by MSigDB [18], and Panther [19].

### Time-dependent differential gene stabilization analysis

At a single time-point of TNF stimulation, mRNA stability for each gene was determined by mRNA expression ratios comparing the RPKMs or normalized read counts in the presence and absence of Act D as follows:

$$S_{t,i}=\frac{{\left(TNF+ActD\right)}_{t,i}}{{TNF}_{t,i}}$$ (1)

where *S*_*t*,*i*_ represents the stability at time point *t* for gene *i*. Formulating stability ratios was informative for determining mRNA stability at individual time-points. However, comparing these ratios between two time-points of TNF stimulation could be challenging to interpret due to the confounding effects of gene expression changes resulting from the time-dependent effects of TNF and Act D. Therefore, we utilized the RiboDiff statistical framework as a tool to determine differentially stable genes between time points of TNF stimulation (S1 Fig). RiboDiff was initially designed for analyzing translational efficiency between conditions using Ribosome Profiling and RNA-seq datasets [20]. Read counts from Ribosome Footprinting are dependent on the read counts from RNAseq in the initial RiboDiff method. A similar dependent relationship is also present in RNA stability data as the (TNF+Act D) condition read counts for a given gene are dependent on the (TNF) condition read counts for this gene. RiboDiff is powered to compare dependent ratios and was utilized to evaluate changes in mRNA stability between two time-points of TNF stimulation. The core of the RiboDiff method is a generalized linear model that approximates the relationship between the TNF and TNF+Act D samples as follows:

$$\log\left(\mu_{TNF,C}^{i}\right)=\beta_{C}^{i}+\beta_{TNF}^{i}$$ (2)

$$\log\left(\mu_{TNF+ActD,C}^{i}\right)=\beta_{C}^{i}+\beta_{TNF+ActD}^{i}+\beta_{\Delta ,C}^{i}$$ (3)

where the expected log read count μ of a gene *i* from the TNF samples [$\log\left(\mu_{TNF,C}^{i}\right)$] is modeled by the contributions of the time condition ($\beta_{C}^{i}$) and the observed RNA-seq read counts ($\beta_{TNF}^{i}$). Similarly, the expected log read count μ of a gene *i* from the TNF+Act D treated samples [$\log\left(\mu_{TNF+ActD,C}^{i}\right)$] is dependent on a time condition ($\beta_{C}^{i}$) and the observed RNA-seq read counts ($\beta_{TNF+ActD}^{i}$). The variable *C* represents either the early (1 hour) TNF stimulation (*C =* 0) or the late (72 hours) TNF stimulation (*C =* 1) time-point. The shared parameter $\beta_{C}^{i}$ that is present in the equations refers to the TNF (Eq 2) and TNF+Act D (Eq 3) conditions. Therefore, the parameter $\beta_{C}^{i}$ represents the shared effect of either the 1h or 72h TNF treatment on the expected log read count. The additional term in Eq 3 (TNF+Act D condition), $\beta_{\Delta ,C}^{i}$, represents the effect of the time parameter on the TNF+Act D treated samples. Thus, genes that demonstrate differential mRNA stability can be tested between the early time-point (1h, *C =* 0) and the late time-point (72h, *C* = 1). Statistical significance for each gene is determined by testing the log-likelihood of the null model ($\beta_{\Delta ,1}^{i}$ = 0) and the alternative model ($\beta_{\Delta ,1}^{i}\neq 0$), which follows a χ^2^ distribution with one degree of freedom. Intuitively, genes that do not demonstrate differential stabilization will fail to reject the null model ($\beta_{\Delta ,1}^{i}$ = 0) as the read count based-ratios between 1h and 72h will not be statistically significant. Statistical significance determined from RiboDiff is reported as FDR adjusted p-values. Lowly expressed genes with normalized read counts less than 10 in the TNF condition were not included in this analysis. For additional details, see S1 Fig that shows in greater detail the graphical model of how the RiboDiff statistical framework was utilized for mRNA stability analysis.

### Multiplex analysis of human cytokines

Magnetic bead-based sandwich immunoassays for cytokines using MILLIPLEX MAP multiplex Human Cytokine Panel 1 (cat #HCYTOMAG-60k; EMD-Millipore Corporation, St. Charles, MO) were performed according to the manufacturer’s instruction. Duplicate wells of cell culture supernatant samples (25 μl) were analyzed by Luminex MagPix (Luminex Corp, Austin, TX). Cytokine concentrations were determined by Luminex Xponent 4.2 and EMD-Millipore Milliplex Analyst v5.1 using 5-p log analysis.

### Statistical analysis of qPCR and multiplex cytokine assay results

Results are expressed as mean ± SEM and GraphPad Prism Analytical Software Version 5 for Windows was used. For statistical analysis of qPCR and multiplex cytokine assay results, the Analysis Of Variance (ANOVA) with Tukey post analysis test was used as appropriate.

## Results

### TNF stabilizes IL-6 mRNA over a prolonged period in RA FLS

**Fig 1:**
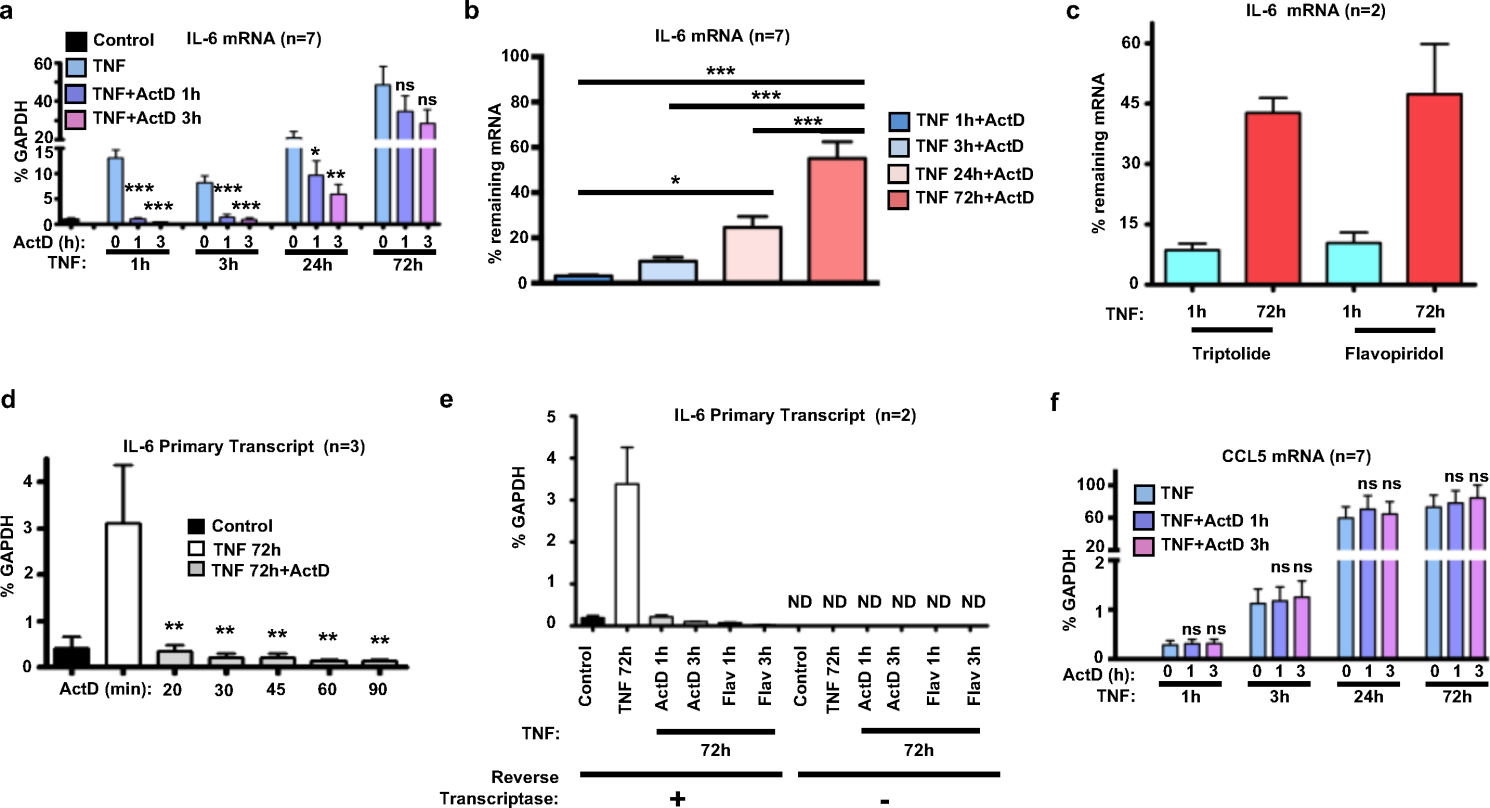
TNF induces late stabilization of IL-6 mRNA in RA FLS. RA FLS were exposed to a single dose of TNF (10 ng/ml) for 1-72h. Subsequently, actinomycin D (Act D; 10 μg/ml) or triptolide (1 μM), or flavopiridol (Flav; 0.5 μM) was added for 1 or 3h and qPCR was used to measure the mRNA levels of IL-6 (a-c), the primary transcripts (PT) of *IL6* (d-e), and the mRNA levels of CCL5 (f). For (b-c), the remaining expression of IL-6 after exposure to inhibitors of transcription (Act D, triptolide, and flavopiridol) was calculated as % of the IL-6 mRNA expressed in the absence of inhibitor at the corresponding TNF-stimulated condition. For (a-b) and (f), cumulative results from 7 independent experiments are shown. For (d-e), FLS were exposed to a single dose of TNF (10 ng/ml) for 72 hours and then inhibitors (Act D or flavopiridol) were added for the indicated time points to block active transcription. Primers specific for the fourth intronic region of *IL6* were designed to capture primary transcripts of *IL6*.Values were normalized relative to GAPDH mRNA and presented as mean ±SEM. GAPDH was considered an appropriate internal control for normalizing qPCR results since TNF stimulation had no impact on expression levels and stability status of GAPDH mRNA (S1 Table). *P* values were calculated by one-way ANOVA and Tukey post-test analysis (* = *p*<0.05, ** = *p*<0.01, *** = *p*<0.001, ns = not significant, and ND = not detected).

We previously showed that a single stimulation of FLS with TNF triggers chromatin remodeling and prolonged transcription of *IL6*, resulting in accumulation of IL-6 mRNA and protein [10]. Here, we wished to test the contribution of mRNA stability to the progressive accumulation of IL-6 mRNA levels over time (Fig 1A, bars 2, 5, 8, and 11; see also [10]). We estimated IL-6 mRNA stability by stimulating cells with TNF for various times, adding RNA polymerase II inhibitor actinomycin D (Act D) to terminate transcription, and measuring IL-6 mRNA 1 or 3 hours later. Consistent with previous reports that IL-6 mRNA is very unstable [21], IL-6 mRNA amounts rapidly decayed to return to baseline 1–3 hours after Act D addition to FLS that had been stimulated with TNF for 1 or 3 hours to induce *IL6* expression (Fig 1A, bars 2–7). Surprisingly, in FLS that had been stimulated with TNF for 24 or 72 hours, IL-6 transcripts persisted after addition of Act D (Fig 1A, bars 8–13). Indeed, this persistence of IL-6 mRNA after Act D addition increased progressively with the time of TNF treatment (Fig 1B). Similar results, suggestive of TNF-induced stabilization of IL-6 mRNA, were observed when triptolide and flavopiridol were used as inhibitors of transcription, instead of Act D (Fig 1C). Notably, Act D, triptolide and flavopiridol arrest transcription via different molecular mechanisms [22]. Whereas Act D binds DNA at the transcription initiation complex and prevents the elongation of RNA chains, triptolide triggers rapid degradation of RNA polymerase II, and flavopiridol inhibits the transcriptional elongation factor P-TEFb. By using primers specific for the fourth intronic region of *IL6* to capture primary transcripts of *IL6*, we have verified that Act D and flavopiridol rapidly induce an almost complete inhibition of transcription even when added during the late phase (72 hours) of TNF stimulation (Fig 1D and 1E). We have excluded the possibility of our primers targeting genomic DNA in addition to primary transcripts since amplification products were not detected when reverse transcriptase was omitted (Fig 1E). These results suggest that IL-6 mRNA becomes stabilized at late time points after TNF stimulation, and such stabilization, coupled with ongoing transcription, can contribute to progressive mRNA accumulation. Similarly to IL-6, CCL5 mRNA is induced by TNF with protracted kinetics in RA FLS [10]. Notably, TNF-induced CCL5 mRNA persisted after Act D addition during both the early and the late phase of TNF stimulation (Fig 1F). These data are in agreement with previous studies showing that CCL5 mRNA is intrinsically stable and displays very slow decay rates [8]. Overall, our observations suggest that in addition to sustained transcription [10], TNF can induce stable transcripts and stabilize previously unstable transcripts to sustain an inflammatory response in FLS.

### Mapping the mRNA stabilome in RA FLS

**Fig 2:**
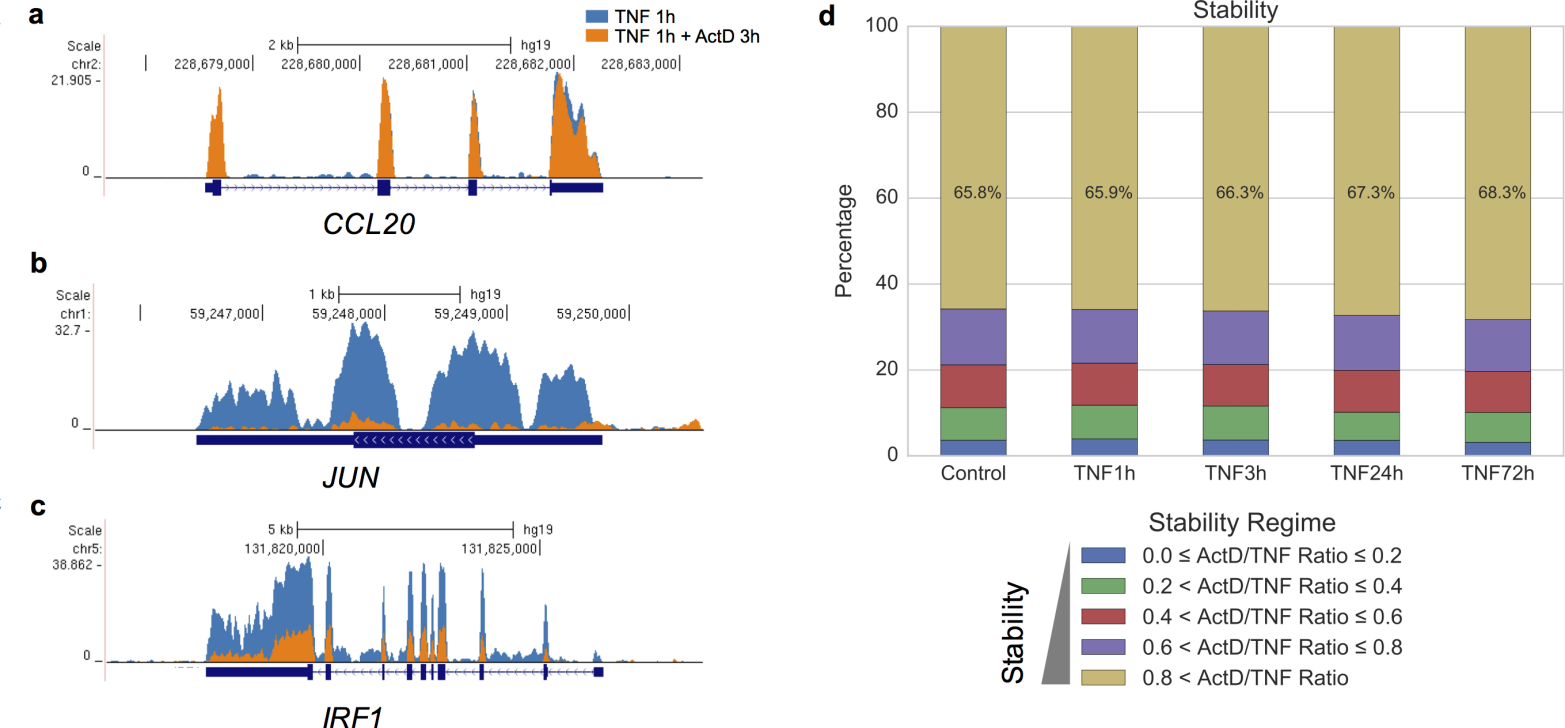
Genome-wide evaluation of mRNA stability states of expressed genes in RA FLS. (a-c), Gene tracks showing sequencing reads from RNA sequencing mapped to *CCL20* (a), *JUN* (b) and *IRF1* (c) genes. The sequencing reads after TNF stimulation for 1 hour without (blue) or with Act D (orange) are shown. (d), Stacked bar graphs illustrating the mRNA stability states of genes expressed in unstimulated (Control) and TNF-stimulated FLS (1, 3, 24 and 72 hours of TNF stimulation). The mRNA stability status was calculated as the ratio of expression levels at the TNF+Act D condition divided to the expression levels at the TNF condition. This ratio ranges from 0 to 1 and classifies genes to a spectrum from very unstable to very stable transcripts. The expressed genes were classified into five groups with distinct stability states and the size of each group is represented as % of total number of expressed genes for each condition.

Aiming to characterize genome-wide the mRNA stability in RA FLS, we performed RNA sequencing and analyzed the effects of Act D in two biological replicates (derived from two different RA patients) of TNF-stimulated FLS. More specifically, RA FLS were stimulated with TNF (10 ng/ml) for 1, 3, 24 or 72 hours and then Act D (10 μg/ml) was added for 3 hours to disrupt transcription. The mRNA stability status was calculated as the ratio of RPKM levels at the TNF+Act D condition divided by the RPKM levels at the TNF condition. This ratio ranges from 0 to 1 and classifies genes along a spectrum ranging from very unstable to very stable transcripts. In Fig 2A–2C), representative gene tracks with differing mRNA stability states are presented. At 1 hour of TNF-stimulation, CCL20 mRNA is very stable (Fig 2A); in contrast JUN mRNA is very unstable (Fig 2B), and IRF1 mRNA displays intermediate stability (Fig 2C). The first conclusion of our analysis was that 65.8–68.3% of expressed transcripts in unstimulated (Control) and TNF-stimulated RA FLS were very stable, namely Act D induced only minor if any downregulation of their mRNA levels (Fig 2D). Furthermore, TNF only minimally altered the relative proportions of transcripts in the various stability classes globally.

### Genome-wide identification of transcripts stabilized by TNF in RA FLS

Inflammation-induced stabilization of arthritogenic transcripts, such as IL-6, might contribute to perpetuation of synovitis. Aiming to identify additional gene-transcripts amenable to stabilization by TNF, we analyzed genome-wide the differential mRNA stability status of genes between the early (1 hour) and the late (72 hours) phases of TNF response. DESeq2 was used to analyze the differential gene expression between TNF and TNF + Act D conditions and calculate statistical significance of differences (adjusted p-values) using two biological replicates (derived from two different RA patients) of RNA sequencing. During the early phase of TNF stimulation transcripts encoded by 2,365 genes were found to be significantly (adjusted p-value <0.1) down-regulated by Act D (S2A Fig). Out of these genes, 2,248 were also expressed at the late phase (72 hours) of TNF stimulation. Comparing the stability state of these expressed genes at 1 and 72 hours of TNF-stimulation, we identified 1,600 genes whose stability was increased during the late phase of TNF responses (stabilized genes) and 648 genes displaying decreased mRNA stability (destabilized genes) (S2B Fig). Notably, the top genes displaying the highest degree of TNF-induced mRNA stabilization were IL-6 and the neutrophil-recruiting chemokines CXCL3, CXCL1, and CXCL8/IL-8 (S2B Fig).

**Fig 3:**
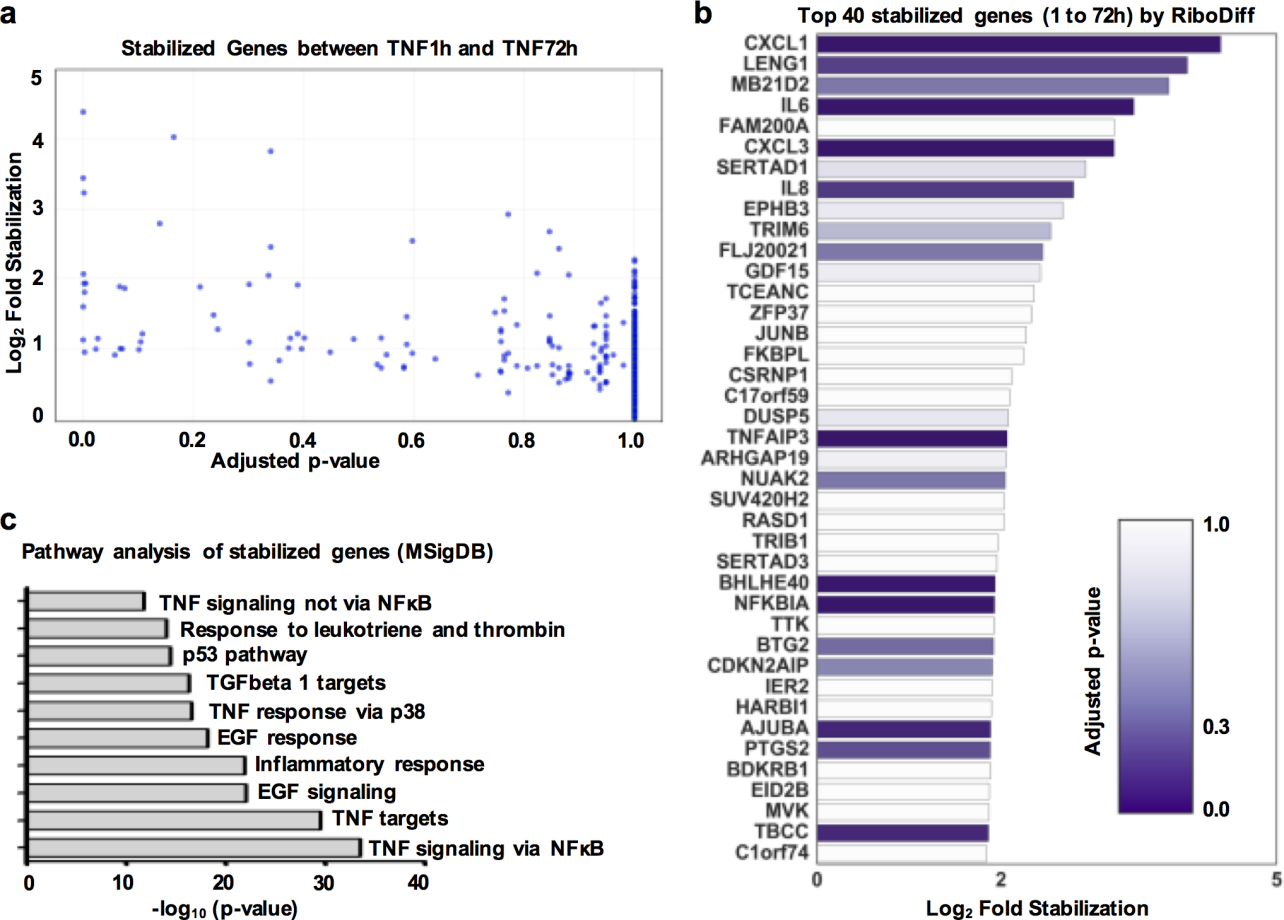
Genome-wide identification of transcripts stabilized by TNF in RA FLS. Two biologic replicates of RA FLS (derived from two different RA patients) were exposed to a single dose of TNF (10 ng/ml) for 1 or 72h. Subsequently, Act D was added for 3h and gene expression was measured by RNA sequencing. The degree of TNF-induced mRNA stabilization was calculated as the log_2_ difference of TNF+Act D/TNF ratio between 1 and 72h of TNF stimulation and the adjusted p values of TNF-induced stabilization were calculated by RiboDiff. (a), Scatter-plot of the genes displaying TNF-induced mRNA stabilization comparing the degree of mRNA stabilization (y axis) to the adjusted p values of the stabilizing effect of TNF (x-axis). (b), The top 40 genes displaying the highest TNF-induced mRNA stabilization ranked by the degree of stabilization. (c), Enriched biological processes identified by GSEA/MSigDB pathway analysis of the top 10% of the genes (n = 593) displaying the highest degree of TNF-induced mRNA stabilization.

We then performed an alternative and more rigorous statistical analysis to identify in a genome-wide manner the genes that were significantly stabilized by TNF. For this purpose we used RiboDiff as a tool to evaluate the stabilization effect of TNF and calculate statistical significance of this effect as described in Methods and S1 Fig. Out of the 12,011 genes that were expressed at both 1 and 72 hours of TNF stimulation, 5,926 genes (49.3%) displayed various degrees of TNF-induced mRNA stabilization. These genes were ranked by the stabilization degree (comparing the mRNA stability at 1 hour of TNF stimulation to the mRNA stability at 72 hours of TNF stimulation) and the adjusted p-values of stabilization as calculated by RiboDiff (Fig 3A). The top 40 genes displaying the highest degree of TNF-induced stabilization are illustrated in Fig 3B. Notably, IL-6, CXCL3, CXCL1, CXCL8/IL-8, and PTGS2 were among these highly stabilized genes. To further validate the stabilizing effect of TNF for these genes that were discovered by the RiboDiff analysis, we performed qPCR measurements of the impact of Act D verifying their substantial TNF-induced mRNA stabilization (S3A Fig). Interestingly, CCL2 mRNA was found by qPCR to be also stabilized by TNF (S3B Fig). In the RiboDiff analysis, CCL2 mRNA displayed a high degree of TNF-induced stabilization, but due to inter-donor variability in the expression levels of CCL2 mRNA, the adjusted p-value did not reach statistical significance. In our qPCR analysis, we have included 7 biological replicates that verify TNF-induced stabilization of CCL2 mRNA.

### Biological consequences of TNF-induced stabilization in RA FLS

To investigate the potential biological consequences of the observed TNF-induced mRNA stabilization, GSEA/MSigDB gene ontology analysis was performed for the top 10% of the stabilized transcripts (593 genes). Interestingly, the stabilized gene-set was highly enriched for a number of potentially pathogenic biologic processes (Fig 3C). It is of note that among the enriched processes are included pathways related to growth factors (“EGF signaling”, “EGF response”, and “TGF beta 1 targets”), cell growth (“p53 pathway”), inflammation (“Inflammatory response”, and “Response to leukotriene and thrombin”), and TNF signaling (“TNF signaling via NF-κB”, “TNF response via p38”, and “TNF signaling not via NF-κB”). Overall, these data suggest that chronic exposure of FLS to TNF increases the mRNA stability status of a wide array of genes, including transcripts with arthritogenic potential.

### TNF-induced molecular mechanisms driving mRNA stabilization

**Fig 4:**
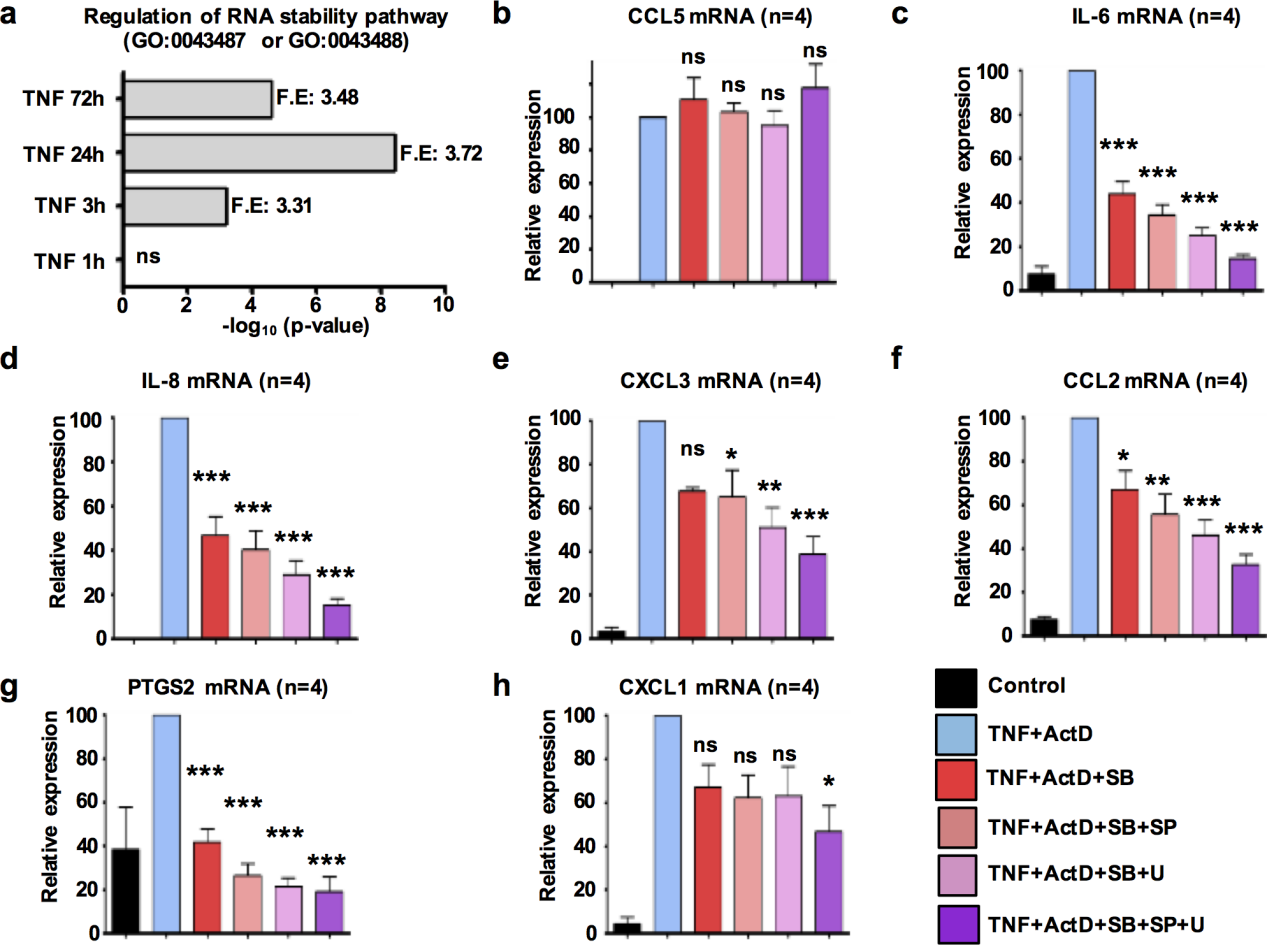
TNF induces expression of mRNA-stabilizing pathways and mRNA stabilization is MAPK-dependent. (a), RNA sequencing was performed in 2 biological replicates (derived from two different RA patients) of TNF-stimulated RA FLS and Panther-Gene Ontology was used to evaluate their enrichment for the biological process “Regulation of RNA stability” (GO:0043487 or GO:0043488). F.E = fold enrichment and ns = not significant. (b-h), RA FLS were exposed to a single dose of TNF (10 ng/ml) for 72h and then Act D (10 μg/ml) was added for 20 mins to block active transcription. Subsequently, the cells were treated for 4h with SB202190 (p38 inhibitor) alone or in various combinations with U0126 (MEK inhibitor) and SP600125 (JNK inhibitor). qPCR was used to measure the mRNA levels of CCL5 (b), IL-6 (c), IL-8 (d), CXCL3 (e), CCL2 (f), PTGS2 (g), and CXCL1 (h). Cumulative results from 4 independent experiments are shown. Values were normalized relative to GAPDH mRNA and presented as mean ±SEM. The mRNA expression at the TNF+Act D condition was set to 100 and the mRNA expression at all the other conditions was calculated as % of the TNF+Act D condition. *P* values were calculated by one-way ANOVA and Tukey post-test analysis (* = *p*<0.05, ** = *p*<0.01, *** = *p*<0.001, and ns = not significant).

Next, we investigated the molecular mechanisms that drive the observed TNF-induced mRNA stabilization. Initially, we tested the hypothesis that TNF induces a transcriptional program that orchestrates late phase stabilization of expressed mRNA transcripts. Using DESeq2, we identified the genes in RA FLS that were regulated by TNF-stimulation in a statistically significant manner (S4 Fig, red represents the genes that were up- or down-regulated by TNF with adjusted p-values<0.1). Notably, pathway analysis using Panther-Gene Ontology Database identified “Regulation of RNA stability pathway” (GO:0043487 and GO:0043488) among the biological processes that were significantly enriched at 3–72 hours of TNF-stimulation (Fig 4A). This observation supports a model where TNF induces a late transcriptional program leading to expression of gene products that stabilize mRNAs in a selective manner.

Several studies suggest that MAPKs are implicated in the regulation of mRNA stability by phosphorylating RNA-binding proteins ([23–26] and summarized in [27,28]). Since TNF-induced activation of MAPKs is a major signaling event downstream of TNF receptors [3], we wished to investigate the potential role of MAPKs in TNF-triggered stabilization of mRNA in our system. RA FLS were exposed to a single dose of TNF (10 ng/ml) for 72 hours. Subsequently, Act D (10 μg/ml) was added for 20 minutes to terminate active transcription. Then, pharmacological inhibitors of p38 (SB202190), JNK (SP600125) and MEK1/2 (U0126) were added in various combinations for 1–4 hours and mRNA levels were measured by qPCR. As expected, pharmacologic inhibition had no effect on the expression levels of CCL5 mRNA (Fig 4B), verifying that the mRNA product of *CCL5* is intrinsically very stable and its stability is not regulated by MAPKs [8]. In contrast, the mRNA levels of stabilized genes, including *IL6* (Fig 4C), *IL8* (Fig 4D), *CXCL3* (Fig 4E), *CCL2* (Fig 4F), *PTGS2* (Fig 4G), and to a lower degree *CXCL1* (Fig 4H), were significantly down-regulated by the pharmacologic inhibitors of MAPKs. Although pharmacologic inhibitors might display specificity issues due to “off-target” effects, these data suggest that the activity of MAPKs during the late phase of TNF responses contributes to the observed TNF-induced stabilization of transcripts.

### Contribution of mRNA stability and TNF-induced stabilization to gene expression levels

**Fig 5:**
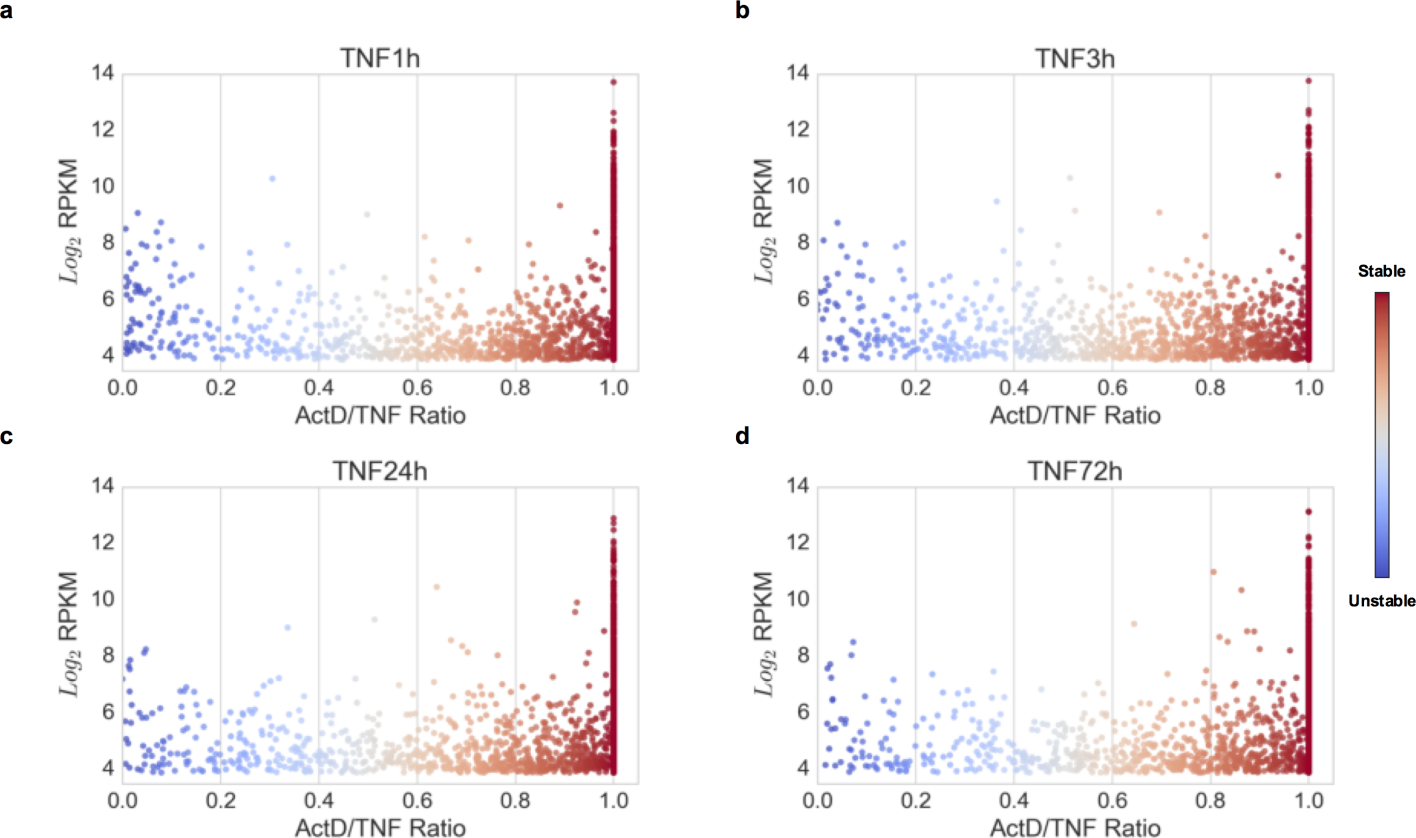
Scatterplots comparing the expression levels to the mRNA stability states of the expressed genes in RA FLS. Two biological replicates of RA FLS (derived from two different RA patients) were exposed to a single dose of TNF (10 ng/ml) for 1, 3, 24, or 72 hours. Subsequently, actinomycin D (Act D, 10μg/ml) was added for 3 hours to block active transcription and gene expression was measured by RNA sequencing. RPKM values were generated using CuffDiff2. The mRNA stability status was calculated genome-wide as the ratio of RPKM levels at the TNF+Act D condition divided to the RPKM levels at the TNF condition. This ratio ranges from 0 to 1 and classifies genes to a spectrum from very unstable to very stable transcripts. The genes expressed at 1 (a), 3 (b), 24 (c), and 72 (d) hours of TNF stimulation were plotted based on their expression levels and the mRNA stability states. Shades of blue represent the region of unstable genes, and shades of red represent the zone of stable genes.

**Fig 6:**
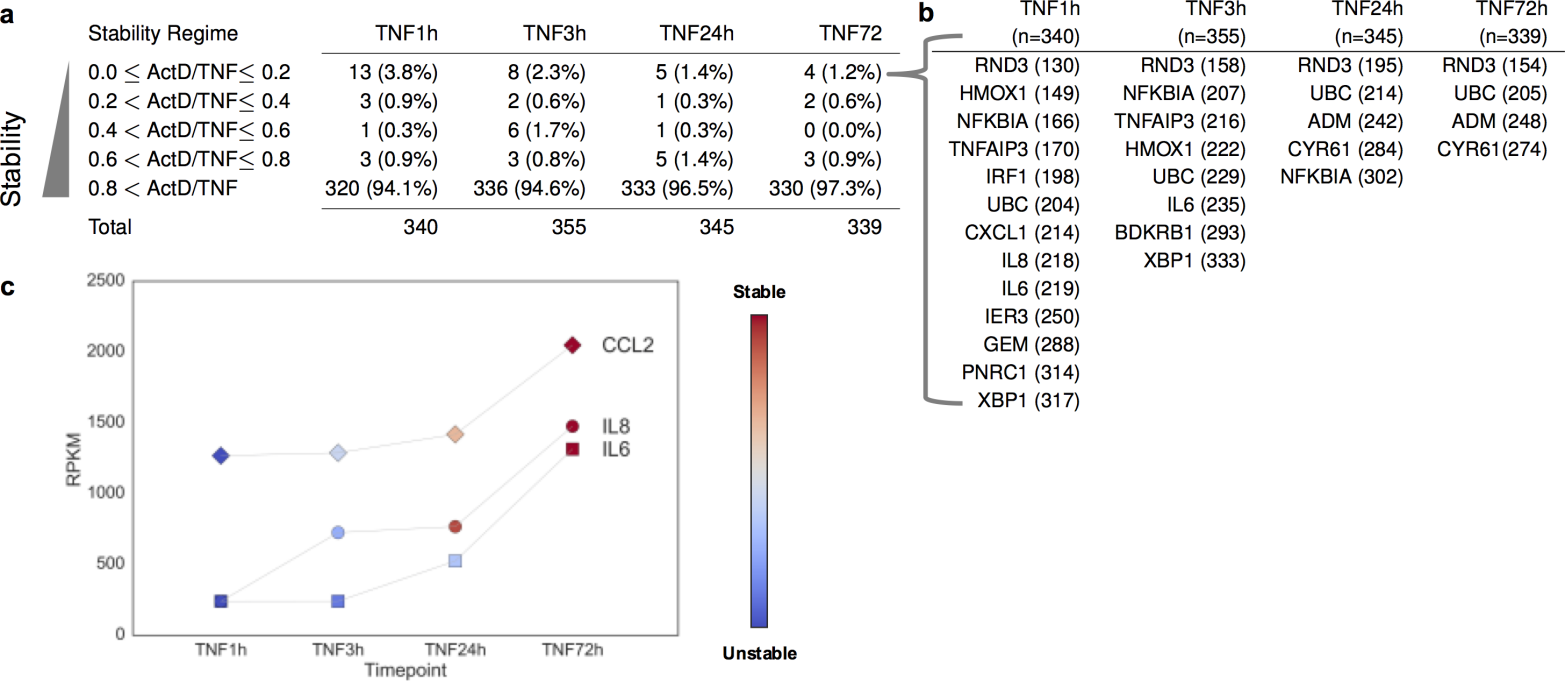
Genome-wide association of the gene expression levels with the mRNA stability states in rheumatoid arthritis (RA) fibroblast-like synoviocytes (FLS). (a), Table of the top 10% of highly expressed genes at 1, 3, 24 and 72 hours of TNF stimulation grouped by their mRNA stability status. The number of genes included in each stability group is presented. (b), Table of the highly expressed genes with very unstable mRNAs. Their ranking by level of expression is presented in parentheses. (c), Kinetics of expression and TNF-induced dynamics of mRNA stability for IL-6, IL-8 and CCL2. Shades of blue represent unstable status and shades of red represent stable status.

During the course of chronic synovitis, RA FLS express high levels of potentially pathogenic transcripts. Although the role of active transcription in driving gene expression is well studied [29], the contribution of mRNA stability to the expression levels of TNF-induced genes is largely unexplored. To investigate this issue, we explored the genome-wide correlation of the expression levels with the mRNA stability states of expressed genes in TNF-stimulated RA FLS (Fig 5, blue dots represent unstable transcripts and red dots represent stable transcripts). A consistent observation for all time points of TNF stimulation (1, 3, 24 and 72 hours) was that among the top 10% of highly expressed genes (>300 genes for each time point) the vast majority (94.1% at 1 hour, 94.6% at 3 hours, and 96.5% at 24 hours, and 97.3% at 72 hours) were genes with very stable mRNAs (Fig 6A). Very few unstable transcripts were included in the list of highly expressed genes (13 genes at 1 hour, 8 genes at 3 hours, 5 genes at 24 hours and 4 genes at 72 hours) (Fig 6A and 6B). These unstable transcripts and their ranking based on their expression level are illustrated in Fig 6B. Upon ranking genes by expression levels, none of the unstable transcripts was ranked higher than 130^th^ (RND3 at 1 hour) out of >300 expressed genes. These data suggest that only genes with very stable transcripts achieve the very top expression levels in RA FLS. At 72 hours, among the very top highly expressed genes are included the highly stabilized IL-6 (ranked 39^th^ by expression level), IL-8 (ranked 32^nd^ by expression level), CCL2 (ranked 20^th^ by expression level), and CXCL1 (ranked 61^st^ by expression level). Monitoring the TNF-induced expression kinetics relative to the TNF-induced stabilization dynamics for IL-6, IL-8 and CCL2 mRNA, it was observed that all three genes achieve their highest expression levels when their transcripts have been stabilized by TNF (Fig 6C, blue represents unstable mRNA and red represents stable mRNA). Overall, these observations suggest that mRNA stability and TNF-induced stabilization represent two mechanisms that potentially contribute, together with active transcription, to the induction and maintenance of high mRNA expression levels.

### Temporal switch in TNF-induced gene response from unstable to stable transcripts

**Fig 7:**
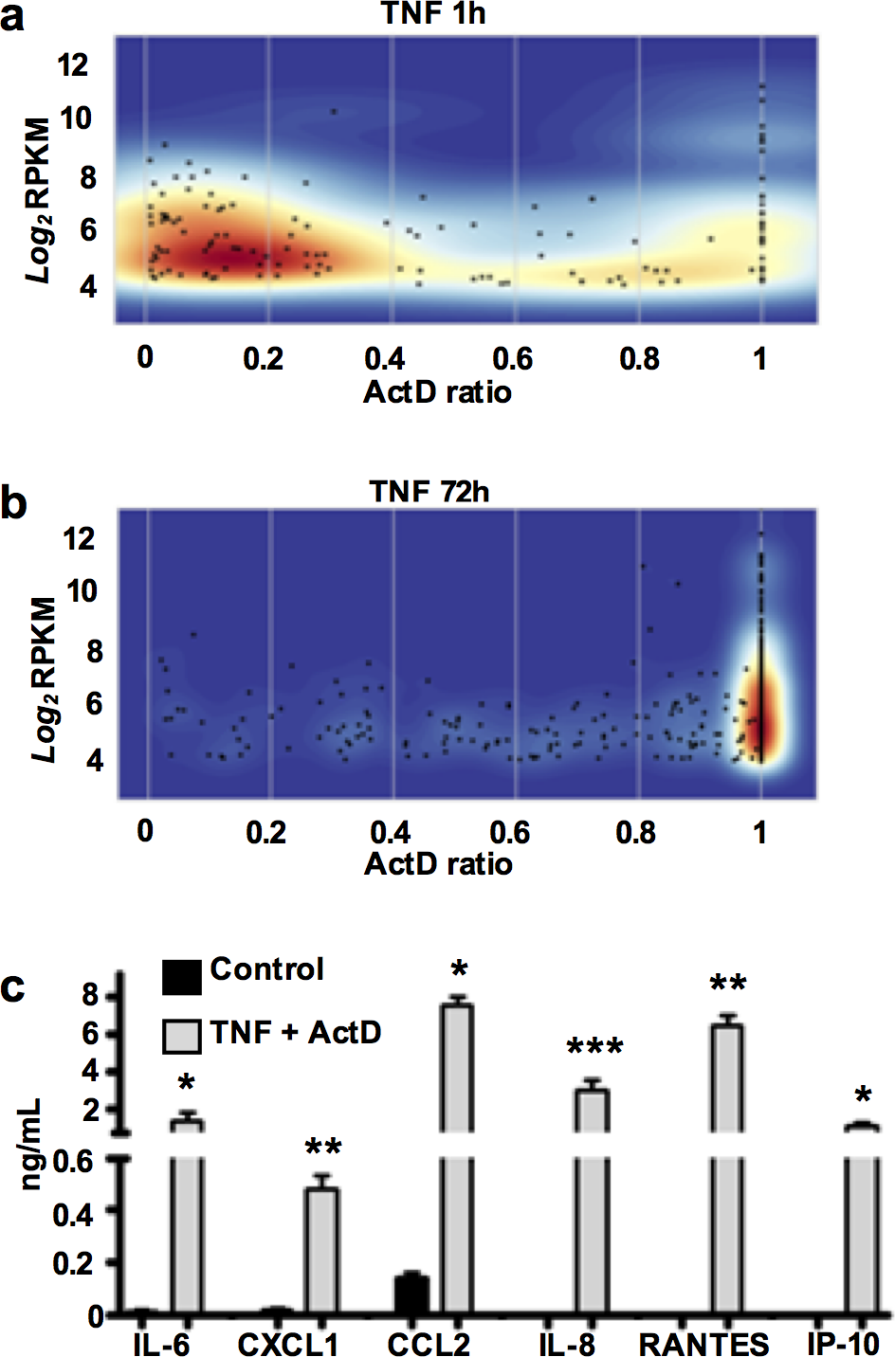
TNF induces a temporal switch from an early program dominated by unstable transcripts to a late program with expansion of the pool of stable transcripts. (a-b), Density-plots illustrating the distribution of TNF-inducible genes (≥2-fold by TNF at 1 (a) and 72h (b)) based on their mRNA stability (shades of red represent areas with the highest numbers of genes (highest density)). The mRNA stability was calculated genome-wide in 2 biologic replicates as the ratio of RPKM levels at the TNF+Act D condition divided to the RPKM levels at the TNF condition. This ratio ranges from 0 (very unstable transcripts) to 1 (very stable transcripts). (c), RA FLS were stimulated with a single dose of TNF (10 ng/ml) for 72 hours and then Act D (10 μg/ml) was added for 1 hour. Subsequently cells were washed and fresh serum-free medium + Act D was replenished. Supernatants were collected 6 hours later and the protein levels of IL-6, CXCL1, CCL2, IL-8, RANTES and IP-10 were measured by magnetic bead-based multiplex immunoassay. Values are the mean ±SEM of three independent experiments. *P* values were calculated by one-way ANOVA and Tukey post-test analysis (* = *p*<0.05, ** = *p*<0.01, and *** = *p*<0.001).

TNF triggers gene expression with a sequential induction order (early, intermediate, and late response genes) [3,7–9]. We wished to investigate the contribution of mRNA stability to the temporal regulation of TNF-induced gene-expression programs in RA FLS. Further analysis of our RNA sequencing experiments revealed that the gene set induced in the early phase TNF response (1 hr) was comprised predominantly of highly unstable transcripts (TNF+Act D/TNF ratio<0.2) (Fig 7A). In sharp contrast, the gene sets induced during the late phase TNF response (3–72 hours) were comprised predominantly of very stable transcripts (TNF+Act D/TNF ratio>0.8) (Fig 7B and S5 Fig). Thus, during the unfolding of a TNF response in RA FLS, there is a temporal switch in the pattern of TNF-induced gene expression from unstable to stable transcripts.

### TNF-induced stable transcripts retain their translational potential in RA FLS

Translation efficiency is an important predictor of protein levels. Notably, cells under stress may display a profound reprogramming of protein expression characterized by translational arrest due to disassembly of translating polysomes that is followed by routing and accumulation of mRNAs into stress granules [30]. In this context, we explored whether the stable transcripts accumulated during the late phase of TNF response were translated into biologically active proteins. RA FLS were stimulated with TNF for 72 hours and then Act D was added for 1 hour to block transcription. Subsequently, cells were washed to remove any protein products that had been secreted by FLS, and fresh serum-free medium with Act D was added for 6 hours. Although active transcription was blocked for at least 7 hours, high levels of IL-6, CXCL1, CCL2, IL-8, RANTES (encoded by *CCL5*) and IP-10 (encoded by *CXCL10*) proteins were measured in culture supernatants (Fig 7C). Overall these data suggest that chronic exposure of RA FLS to TNF results in progressive accumulation of very stable arthritogenic transcripts that retain their potential to be translated into proteins.

### Relationship of mRNA stability status and TNF-induced expression kinetics

**Fig 8:**
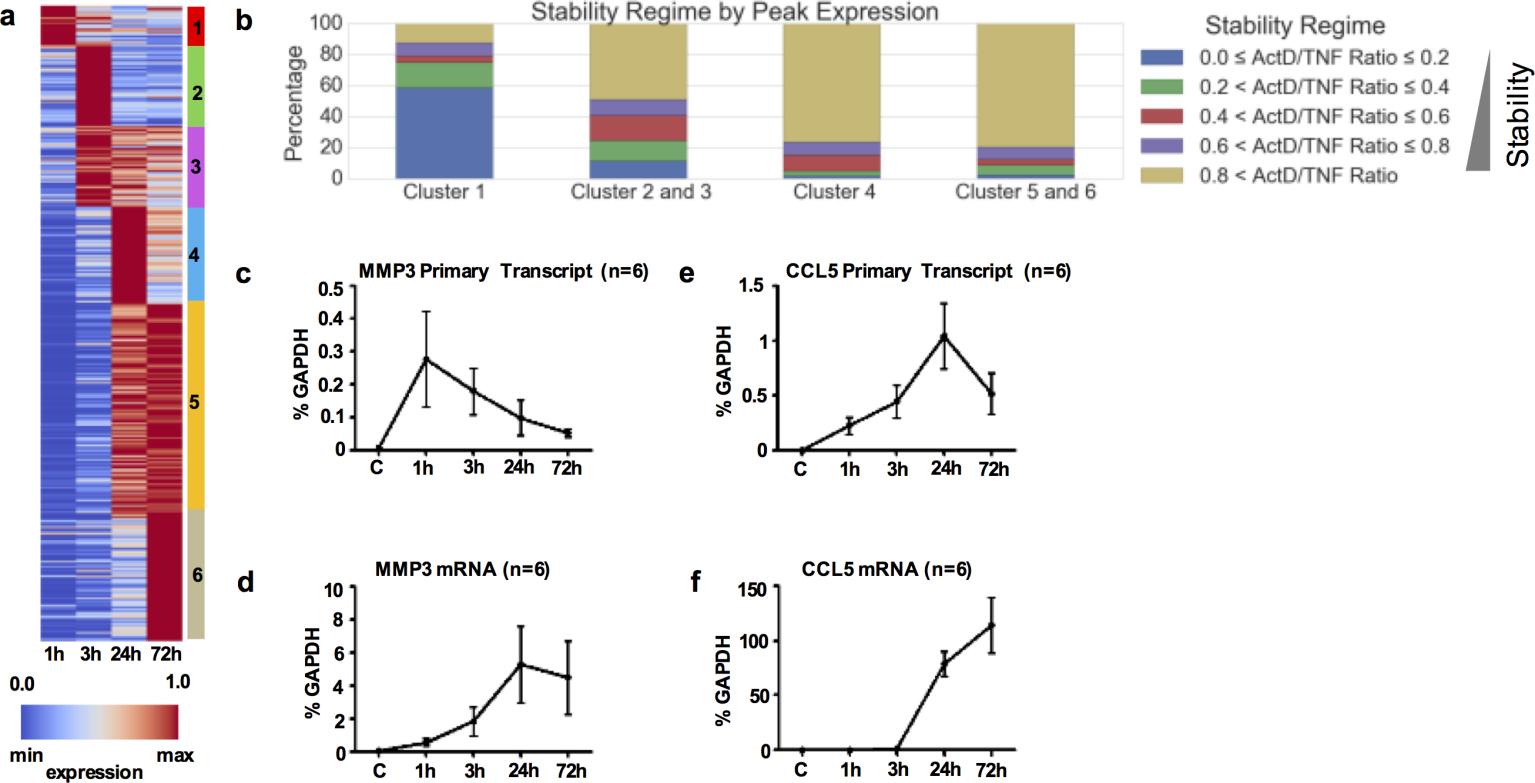
Association of expression kinetics with mRNA stability states of TNF-inducible genes in RA FLS. For (a-b), two biological replicates of RA FLS (derived from two different RA patients) were exposed to a single dose of TNF (10 ng/ml) for 1-72h. Subsequently, Act D (10 μg/ml) was added for 3h and gene expression was measured by RNA sequencing. 386 genes were identified as highly induced (≥5-fold) by TNF at any time point and were clustered into 6 clusters with distinct kinetics of peak expression. (a), Heatmap illustrating the expression kinetics of the 6 clusters (red represents the maximum and blue the minimum expression level across the lane). (b), Stacked bar graphs illustrating the stability states of genes for Cluster 1, Clusters 2 &3, Cluster 4, and Clusters 5 & 6. For (c-f), RA FLS were exposed to a single dose of TNF (10 ng/ml) for 1–72 hours. Primers specific for the eighth intronic region of *MMP3* and for the first intronic region of *CCL5* were designed to capture primary transcripts (PT) of *MMP3* and *CCL5*. qPCR was used to measure the levels of PT and total mRNA of MMP3 (c-d) and CCL5 (e-f). Cumulative results from six independent experiments are shown. Values were normalized relative to mRNA for GAPDH and are presented as mean ±SEM.

TNF-inducible genes can be classified in different groups by the timing of peak expression (early or delayed) and by kinetics of expression (transient or sustained). We analyzed the time course of TNF-induced gene expression in our experimental system focusing on 386 genes that were identified as highly induced by DESeq2 (≥5-fold at 1, or 3, or 24, or 72 hours of TNF-stimulation). Hierarchical clustering of the gene expression values led to the classification of highly induced genes into 6 clusters with distinct timing of peak gene expression and diverse expression duration kinetics (Fig 8A). Cluster 1 represents the early-transient program with peak expression at 1 hour (S6A Fig). Clusters 2 and 3 include genes that peak at 3 hours (S6B Fig), cluster 4 represents the intermediate program including genes with expression peak at 24 hours (S6C Fig), and clusters 5 and 6 include primarily genes with continuously rising expression kinetics that peak at 72 hours (S6D Fig). Next, we explored the potential relationship of the mRNA stability status with the expression kinetics of TNF-inducible genes in RA FLS. Notably, the early-transient program (Cluster 1) is highly enriched with very unstable transcripts (TNF+Act D/TNF ratio<0.2, blue in the first bar of Fig 8B), whereas all the other clusters are dominated by genes with very stable mRNAs (TNF+Act D/TNF ratio>0.8, light brown, Fig 8B). These observations suggest a potential contribution of mRNA stability to the expression kinetics of TNF-inducible genes in RA FLS and support a model where transcript instability contributes to transient expression kinetics, whereas stability contributes to more protracted kinetics.

To further explore this concept, we monitored in parallel the TNF-induced kinetics of total mRNA expression and active transcription (measured by the expression of primary transcripts) of very stable transcripts, such as MMP3 and CCL5. Interestingly, both genes displayed late peaks and protracted expression kinetics, but the kinetics of active transcription were discordant with the total mRNA expression kinetics (Fig 8C–8F). For MMP3, the rate of active transcription dropped gradually after 1 hour (Fig 8C). Despite this drop, the total mRNA continued accumulating and peaked at 24 hours (Fig 8D). A similar pattern was observed for CCL5, with primary transcripts dropping at the time of peak of total mRNA expression (Fig 8E and 8F). The observed discordance in kinetics between active transcription and total mRNA expression suggests that, for very stable transcripts, even very low or decreasing rates of transcription may be sufficient to support continuously rising expression.

## Discussion

Is the mRNA degradation rate per gene constant (‘Constant degradation hypothesis’) or is it changing over time (‘Varying degradation hypothesis’)? Next generation sequencing and metabolic labeling of RNA, combined with computational modeling have resolved the debate by providing evidence that favors the varying degradation hypothesis in T-lymphocytes, dendritic cells, and foreskin fibroblasts [31–33]. To our knowledge, the current study is the first addressing this issue on a genome-wide level in RA FLS. We discovered that TNF induces remarkable stabilization of several hundred transcripts during the late phase of cellular response to TNF. Notably, the list of stabilized transcripts included genes with well-known pathogenic potential such as IL-6, PTGS2, and the chemokines CXCL1, CXCL3, CXCL8/IL-8 and CCL2. Gene ontology analysis revealed additional intriguing biological processes related to growth factor biology and cell growth to be enriched in the stabilized transcripts. Furthermore, we found that the gradual stabilization of IL-6, CXCL8/IL-8 and CCL2 transcripts was synchronous with the continuously rising expression levels, suggesting a potential contribution of inflammation-induced transcript stabilization to the protracted expression kinetics. Overall, these observations support a model where TNF-induced mRNA stabilization operates in concert with inflammation-induced transcription resulting in accumulation of transcripts in RA FLS that fuel and perpetuate RA synovitis.

A plausible explanation for TNF-triggered stabilization of transcripts is the induction of molecular pathways that finally inhibit the mRNA degradation machinery [27]. Notably, we found that TNF induces in RA FLS a gene expression program enriched in the biologic process named ‘regulation of RNA stability’ (GO:0043487 and GO:0043488; Fig 4A). One alternative explanation for the observed mRNA stabilization is that TNF regulates the microRNA pathway in RA FLS. microRNAs target messenger RNAs via limited base pairing and recruit the degradation machinery triggering mRNA decay [34]. A second alternative scenario for the TNF-induced stabilization could be the saturation of the degradation machinery in RA FLS due to intracellular accumulation of transcripts during the late phase of TNF response [33]. Several observations in our experimental system do not support the scenario of overwhelmed degradation machinery: (i) we have not observed any dramatic increase in the total RNA concentration by TNF stimulation; (ii) numerous unstable transcripts retained very high mRNA decay rates during the late phase of TNF response; (iii) if the degradation machinery was saturated we should not see the prompt destabilizing effect by pharmacologic inhibition of MAPKs (Fig 4C–4H). A third alternative interpretation for the TNF-induced transcript stabilization in RA FLS could be sequestration of transcripts within stress granules (SG) or processing bodies (PB) [30]. Although we cannot exclude this possibility, transcripts packaged into SG or PB are expected to be translationally silent [30], whereas in our system the stabilized IL-6, CXCL1, CCL2 and CXCL8/IL-8 mRNAs were translated with efficiency into secreted protein products (Fig 7C).

A very interesting finding of our study was the discovery of a temporal switch in the stability states of TNF-induced transcripts in RA FLS. Unstable transcripts dominate the early response to TNF, while during the late TNF response the gene expression program is outbalanced by very stable transcripts (Fig 7A and 7B and S5 Fig). Whereas transient stabilization of select transcripts can slightly extend transient early expression kinetics in other cell types such as macrophages, to our knowledge extended stabilization that markedly switches expression kinetics to a sustained pattern is unprecedented. In the context of RA synovitis, where there is long-term exposure of FLS to TNF and other inflammatory mediators, our data are in accord with reports showing that FLS accumulate high levels of pathogenic transcripts, and in the current study we have found to be stable and stabilized. Our findings about the TNF-induced dynamics in mRNA stabilome in RA FLS suggest that targeting of pathways that stabilize TNF-induced mRNAs can be used to modulate FLS activation and production of arthritogenic mediators in settings of synovial inflammation. Inhibition of TNF-induced mRNA-stabilizing pathways may offer new opportunities for therapeutic intervention in RA synovitis. The results of the current study provide a new view on RA pathogenesis, and together with the data from our previous reports [10,11] support a model where sustained signaling, long-standing chromatin remodeling and mRNA stabilization, all together contribute to the aggressive FLS behavior observed during the chronic course of RA synovitis.

## Supporting information

S1 Table Expression levels and stability of GAPDH mRNA do not change by TNF stimulation. (PDF)

S1 FigGraphical model depicting RiboDiff Method for studying mRNA Stability.The graphical model explains the RiboDiff method applied to evaluate time-dependent, TNF induced changes in mRNA stability. Gray circles depict observed variables: top grey circle indicates the time-point of TNF stimulation (*C* which in the current study is either 1h or 72h); left grey circle is for a given gene *i* the read count from the TNF condition at the time-point *C* (*y*^*i*^_*TNF*,*C*_); right grey circle is for a given gene *i* the read count from the TNF+Act D condition at the time-point *C* (*y*^*i*^_*TNF+ActD*,*C*_). The *r* term denotes replicates for the TNF and TNF+Act D libraries. Empty circles represent unobserved variables that include the dispersion parameters (denoted by *K)*, which are estimated by performing a gamma regression on the raw dispersions, and normalized counts (denoted by μ) that are estimated independently for the TNF and TNF+Act D libraries. Black squares are equations that estimate the expected log read count and model the relationship between TNF and TNF+Act D read count abundances. The $\beta_{C}^{i}+\beta_{TNF}^{i}$ term represents the expected read count for a gene *i*, under time condition *C* for the TNF library. The $\beta_{C}^{i}+\beta_{TNF+ActD}^{i}+\beta_{\Delta ,C}^{i}$ term represents the expected read count for a gene *i*, under time condition *C* for the TNF+Act D library. The term $\beta_{C}^{i}$ represents the shared effect of either the 1h or 72h TNF treatment on the read counts. The term $\beta_{\Delta ,C}^{i}$ represents for a gene *i* the differential effect of the time condition *C* on the TNF+Act D library. RiboDiff tests the significance of the $\beta_{\Delta ,C}^{i}$ term for each gene (test indicated by the dashed arrow).(PDF)

S2 Fig Genome-wide identification of transcripts amenable to TNF-induced mRNA stabilization in RA FLS. (a), Graph illustrating genes expressed at 1 hour of TNF-stimulation and downregulated by actinomycin D (Act D). Gene expression was measured by RNA sequencing. Genes were filtered for expression (raw reads > 100). Differential testing was performed using DESeq2 for the TNF 1h condition against the TNF + Act D condition using two biological replicates. Significance from DESeq2 is presented as the adjusted p-values < 0.1 (red dots) and ≥ 0.1 (grey dots). The x-axis represents the expression level at TNF 1 hour as the mean of normalized counts from two biological replicates. The y axis represents the log_2_ fold change of the TNF + Act D normalized counts over the TNF 1h normalized counts. (b) Of the significantly downregulated genes depicted in (a), 2248 were also expressed at the TNF 72h time point. Differential testing of the TNF72h against the TNF72h with Act D was performed. The corresponding log_2_ fold change at TNF72h (y-axis) is plotted against the log_2_ fold change at TNF1h (x-axis). Genes with an increased log_2_ fold change (orange) represent genes that were stabilized at 72 hours (compared to 1 hour). Destabilized genes demonstrate reduced log_2_ fold change at 72 hours (compared to 1 hour) (blue).
(PDF)

S3 Fig TNF induces late stabilization of IL-8, CXCL1, CXCL3, PTGS2, and CCL2 mRNA in RA FLS. RA FLS were exposed to a single dose of TNF (10 ng/ml) for 1, 3, 24 and 72 hours. Subsequently, actinomycin D (Act D, 10 μg/ml) was added for 1 or 3 hours to block active transcription. Real-time quantitative reverse transcription polymerase chain reaction was used to measure the mRNA levels of IL-8, CXCL1, CXCL3, PTGS2 (a), and CCL2 (b) mRNA. Cumulative results from seven independent experiments are shown. Values were normalized relative to mRNA for GAPDH and are presented as mean ±SEM. *P* values were calculated by one-way ANOVA and Tukey post-test analysis (* = *p*<0.05, ** = *p*<0.01, *** = *p*<0.001, and ns = not significant).
(PDF)

S4 Fig TNF regulated gene expression programs in RA FLS. (a-d), Bland-Altman plots of TNF regulated genes at 1 (a), 3 (b), 24 (c) and 72 (d) hours of TNF-stimulation. The y axis represents the log_2_ fold change (up- or down-regulation) compared to unstimulated cells (Control). The x axis represents expression level as normalized counts (average from two biological replicates). DESeq2 was used to analyze the TNF-induced differential gene expression and to calculate statistical significance. Red color visualizes genes up- or down-regulated by TNF to a statistically significant degree (adjusted p-value< 0.1).
(PDF)

S5 Fig Density-plots illustrating the mRNA stability states of genes induced by TNF at 3 and 24 hours in RA FLS. RA FLS were exposed to a single dose of TNF (10 ng/ml) for 3 or 24 hours. Subsequently, actinomycin D (Act D, 10 μg/ml) was added for 3 hours to block active transcription. Gene expression was measured by RNA sequencing in two biological replicates and RPKM values were generated using CuffDiff2. The mRNA stability status was calculated genome-wide as the ratio of RPKM levels at the TNF+Act D condition divided to the RPKM levels at the TNF condition. This ratio ranges from 0 to 1 and classifies genes to a spectrum from very unstable to very stable transcripts. Genes induced ≥2-fold by TNF at 3 hours (a) and 24 hours (b), were plotted comparing their expression levels (y axis; log_2_ RPKM) to their mRNA stability states (x axis; TNF+Act D/TNF ratio).
(PDF)

S6 Fig Expression kinetics of TNF-inducible genes in RA FLS. Two biological replicates of RA FLS (derived from two different RA patients) were exposed to TNF (10ng/ml) for 1-72h and gene expression was measured by RNA sequencing. 386 genes were identified as highly induced (≥5-fold) by TNF at any time point and were clustered into 6 clusters with distinct kinetics of peak expression. For (a-d), line graphs of mean expression (read counts) at 0-72h of TNF stimulation for each cluster.
(PDF)
