# Supplementary material for: Tumor Necrosis Factor dynamically regulates the mRNA stabilome in rheumatoid arthritis fibroblast-like synoviocytes
Source: PLoS One. 2017 Jul 14;12(7):e0179762. doi: 10.1371/journal.pone.0179762 (PMC5510804; doi:10.1371/journal.pone.0179762)
Supplement: S1 Table — (PDF) [file pone.0179762.s001.pdf]

	GAPDH						
Condition	Donor 1	Donor 2	Donor 3	Donor 4	Donor 5	Donor 6	Donor 7
Control	18.81	18.16	17.89	19.63	18.37	18.00	17.50
Control ActD 1h	18.62	18.29	17.90	19.91	18.31	18.00	17.60
Control ActD 3h	18.60	18.05	17.79	18.90	18.30	18.10	17.70
TNF 1h	19.71	18.50	18.06	19.96	18.93	18.10	17.60
TNF 1h ActD 1h	20.29	19.11	18.60	19.17	19.58	18.10	17.70
TNF 1h ActD 3h	19.53	19.37	18.77	18.43	19.64	18.20	17.80
TNF 3h	20.57	18.93	18.70	18.39	19.31	18.00	17.80
TNF 3h ActD 1h	19.37	18.44	18.34	18.51	19.52	18.20	17.80
TNF 3h ActD 3h	18.60	18.45	17.94	19.21	18.67	18.20	17.90
TNF 24h	18.90	18.53	17.50	18.75	19.21	18.00	17.50
TNF 24h ActD 1h	18.85	18.91	17.42	18.39	19.45	18.00	17.70
TNF 24h ActD 3h	19.62	18.97	18.74	19.63	19.69	18.10	17.90
TNF 72h	19.61	18.61	18.38	19.69	19.21	18.30	17.50
TNF 72h ActD 1h	19.39	18.15	18.47	19.56	18.83	18.20	17.70
TNF 72h ActD 3h	19.76	18.08	17.97	20.34	18.87	18.30	17.90

S1 Table: Expression levels and stability of GAPDH mRNA do not change by TNF stimulation.
Expression levels provided in units of average cycle thresholds (Ct Values). For each condition and donor, average taken from n=3 replicates.
