# Supplementary figures and images for: Tumor Necrosis Factor dynamically regulates the mRNA stabilome in rheumatoid arthritis fibroblast-like synoviocytes
Source: PLoS One. 2017 Jul 14;12(7):e0179762. doi: 10.1371/journal.pone.0179762 (PMC5510804; doi:10.1371/journal.pone.0179762)

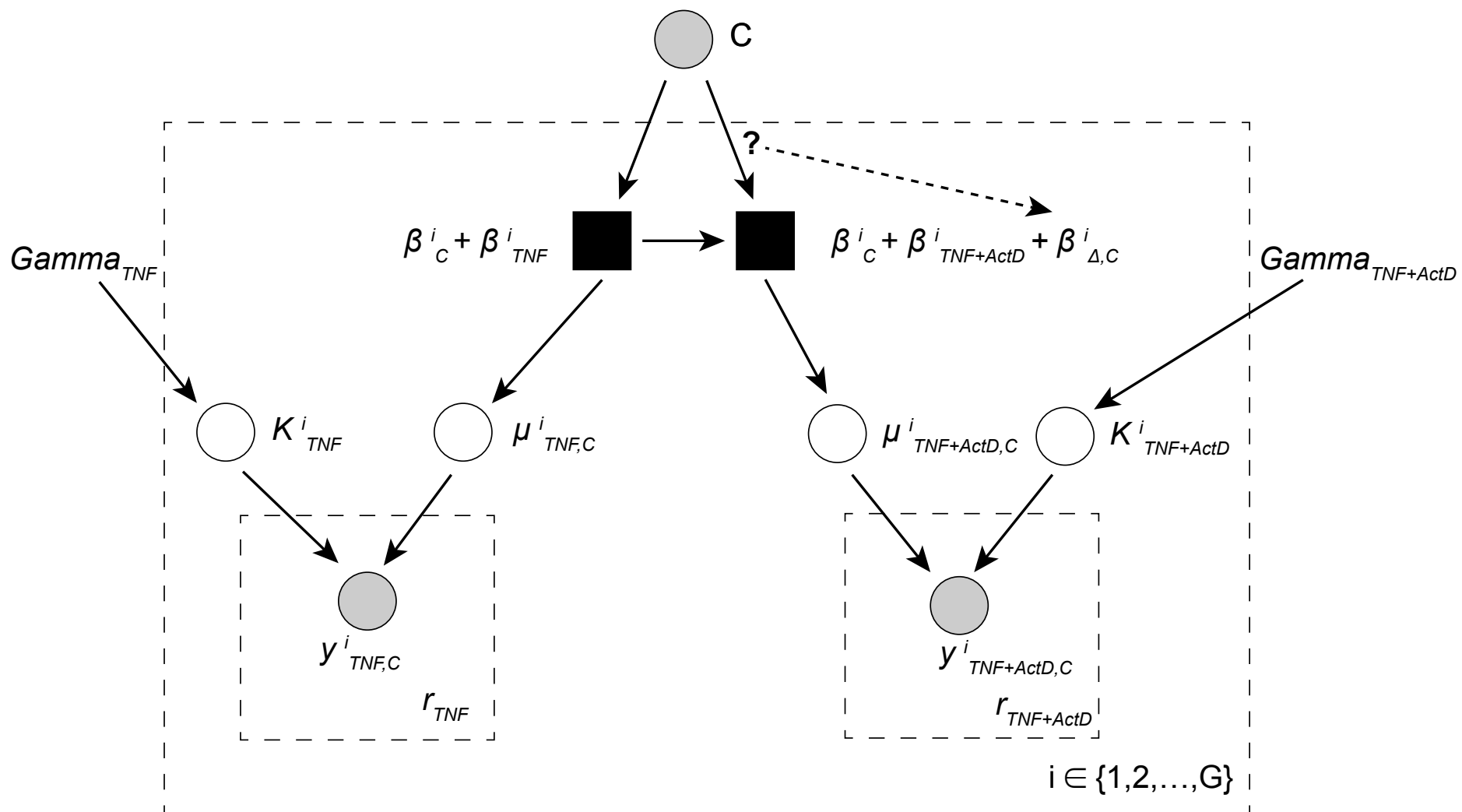

S1 Fig

Supplement: S1 Fig — The graphical model explains the RiboDiff method applied to evaluate time-dependent, TNF induced changes in mRNA stability. Gray circles depict observed variables: top grey circle indicates the time-point of TNF stimulation (C which in the current study is either 1h or 72h); left grey circle is for a given gene i the read count from the TNF condition at the time-point C (yiTNF,C); right grey circle is for a given gene i the read count from the TNF+Act D condition at the time-point C (yiTNF+ActD,C). The r term denotes replicates for the TNF and TNF+Act D libraries. Empty circles represent unobserved variables that include the dispersion parameters (denoted by K), which are estimated by performing a gamma regression on the raw dispersions, and normalized counts (denoted by μ) that are estimated independently for the TNF and TNF+Act D libraries. Black squares are equations that estimate the expected log read count and model the relationship between TNF and TNF+Act D read count abundances. The βCi+βTNFi term represents the expected read count for a gene i, under time condition C for the TNF library. The βCi+βTNF+ActDi+βΔ,Ci term represents the expected read count for a gene i, under time condition C for the TNF+Act D library. The term βCi represents the shared effect of either the 1h or 72h TNF treatment on the read counts. The term βΔ,Ci represents for a gene i the differential effect of the time condition C on the TNF+Act D library. RiboDiff tests the significance of the βΔ,Ci term for each gene (test indicated by the dashed arrow). (PDF) [file pone.0179762.s002.pdf]

**a**

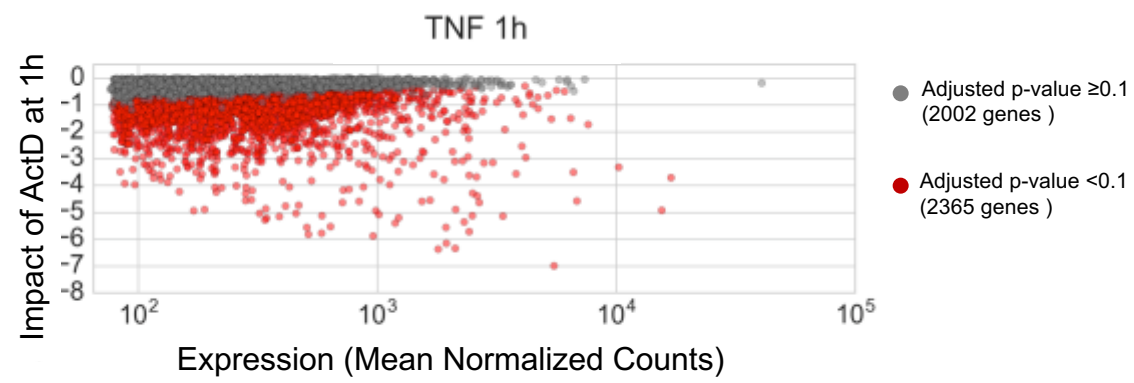

**b**

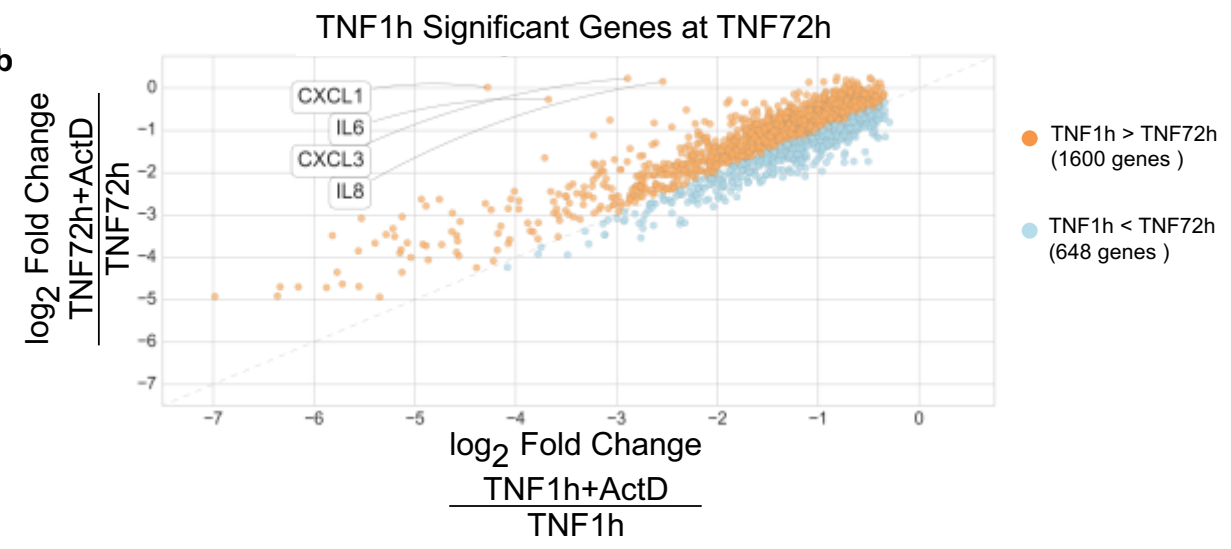

Supplement: S2 Fig — (a), Graph illustrating genes expressed at 1 hour of TNF-stimulation and downregulated by actinomycin D (Act D). Gene expression was measured by RNA sequencing. Genes were filtered for expression (raw reads > 100). Differential testing was performed using DESeq2 for the TNF 1h condition against the TNF + Act D condition using two biological replicates. Significance from DESeq2 is presented as the adjusted p-values < 0.1 (red dots) and ≥ 0.1 (grey dots). The x-axis represents the expression level at TNF 1 hour as the mean of normalized counts from two biological replicates. The y axis represents the log2 fold change of the TNF + Act D normalized counts over the TNF 1h normalized counts. (b) Of the significantly downregulated genes depicted in (a), 2248 were also expressed at the TNF 72h time point. Differential testing of the TNF72h against the TNF72h with Act D was performed. The corresponding log2 fold change at TNF72h (y-axis) is plotted against the log2 fold change at TNF1h (x-axis). Genes with an increased log2 fold change (orange) represent genes that were stabilized at 72 hours (compared to 1 hour). Destabilized genes demonstrate reduced log2 fold change at 72 hours (compared to 1 hour) (blue). (PDF) [file pone.0179762.s003.pdf]

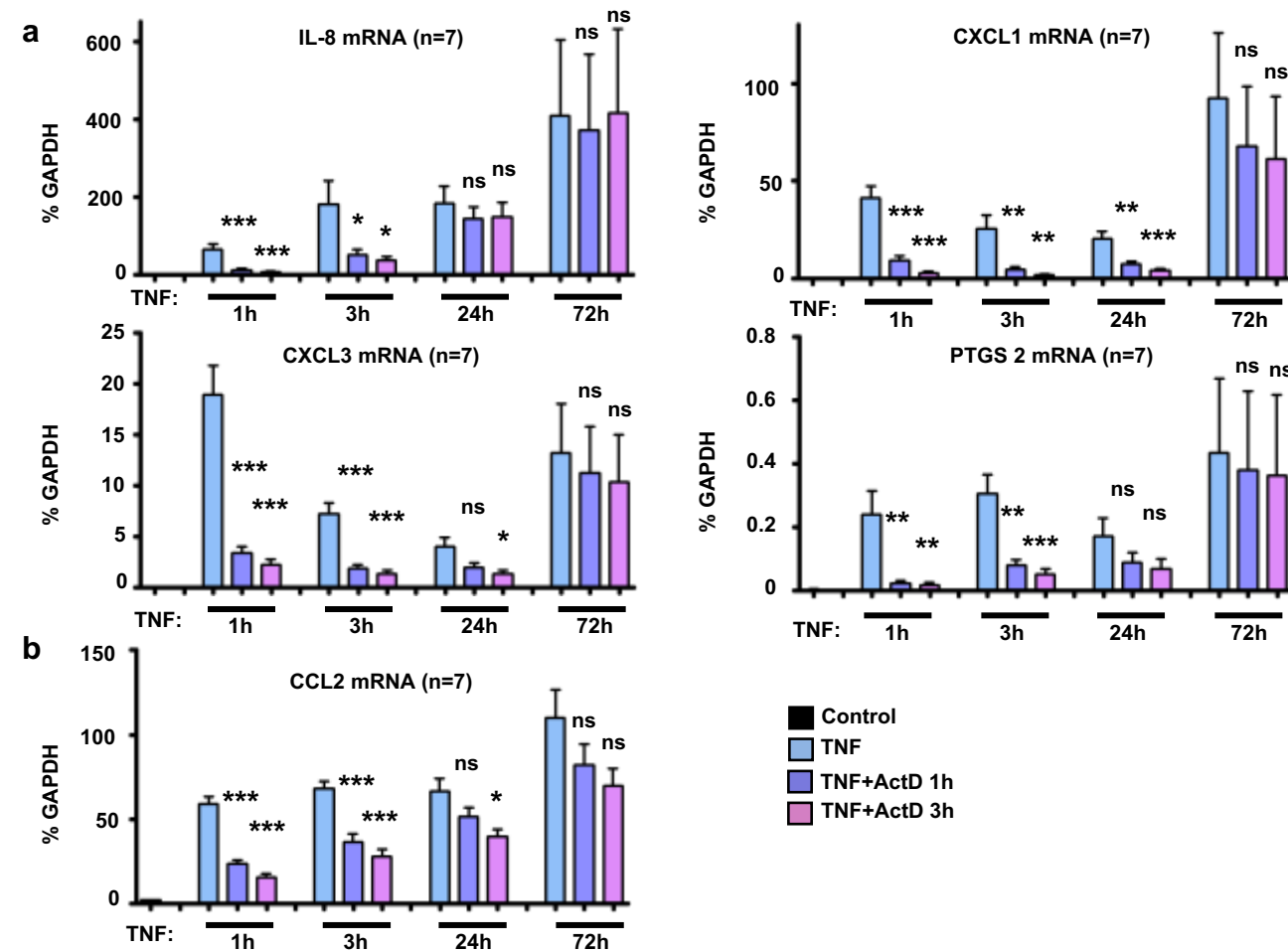

Supplement: S3 Fig — RA FLS were exposed to a single dose of TNF (10 ng/ml) for 1, 3, 24 and 72 hours. Subsequently, actinomycin D (Act D, 10 μg/ml) was added for 1 or 3 hours to block active transcription. Real-time quantitative reverse transcription polymerase chain reaction was used to measure the mRNA levels of IL-8, CXCL1, CXCL3, PTGS2 (a), and CCL2 (b) mRNA. Cumulative results from seven independent experiments are shown. Values were normalized relative to mRNA for GAPDH and are presented as mean ±SEM. P values were calculated by one-way ANOVA and Tukey post-test analysis (* = p<0.05, ** = p<0.01, *** = p<0.001, and ns = not significant). (PDF) [file pone.0179762.s004.pdf]

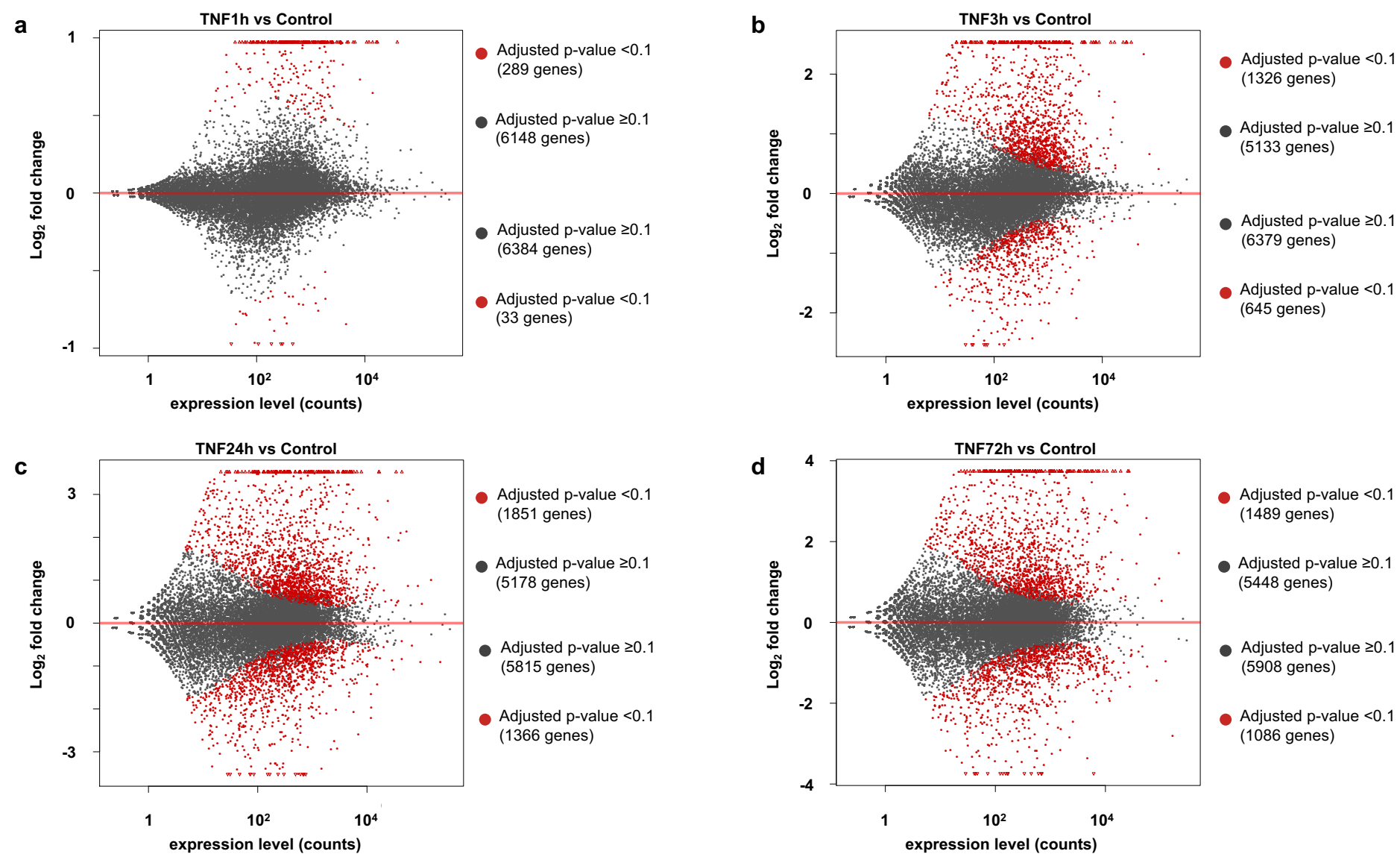

Supplement: S4 Fig — (a-d), Bland-Altman plots of TNF regulated genes at 1 (a), 3 (b), 24 (c) and 72 (d) hours of TNF-stimulation. The y axis represents the log2 fold change (up- or down-regulation) compared to unstimulated cells (Control). The x axis represents expression level as normalized counts (average from two biological replicates). DESeq2 was used to analyze the TNF-induced differential gene expression and to calculate statistical significance. Red color visualizes genes up- or down-regulated by TNF to a statistically significant degree (adjusted p-value< 0.1). (PDF) [file pone.0179762.s005.pdf]

**a**

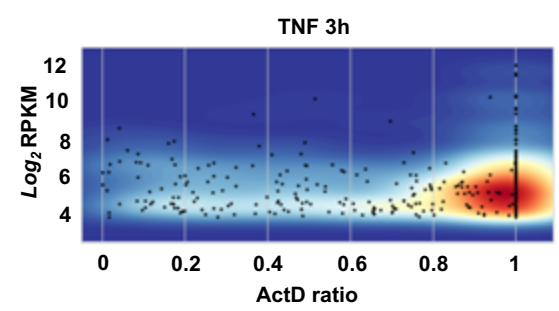

**b**

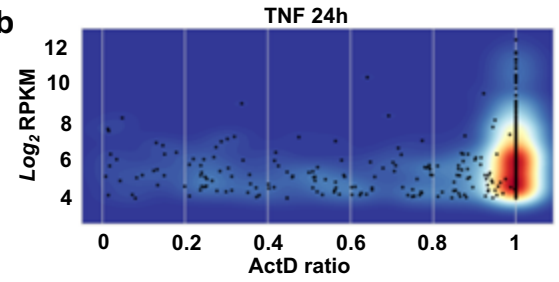

Supplement: S5 Fig — RA FLS were exposed to a single dose of TNF (10 ng/ml) for 3 or 24 hours. Subsequently, actinomycin D (Act D, 10 μg/ml) was added for 3 hours to block active transcription. Gene expression was measured by RNA sequencing in two biological replicates and RPKM values were generated using CuffDiff2. The mRNA stability status was calculated genome-wide as the ratio of RPKM levels at the TNF+Act D condition divided to the RPKM levels at the TNF condition. This ratio ranges from 0 to 1 and classifies genes to a spectrum from very unstable to very stable transcripts. Genes induced ≥2-fold by TNF at 3 hours (a) and 24 hours (b), were plotted comparing their expression levels (y axis; log2 RPKM) to their mRNA stability states (x axis; TNF+Act D/TNF ratio). (PDF) [file pone.0179762.s006.pdf]

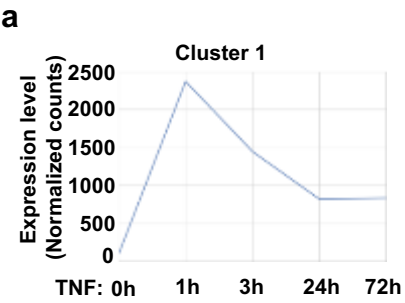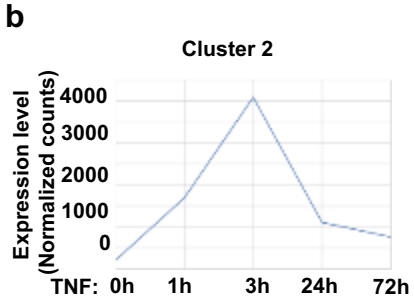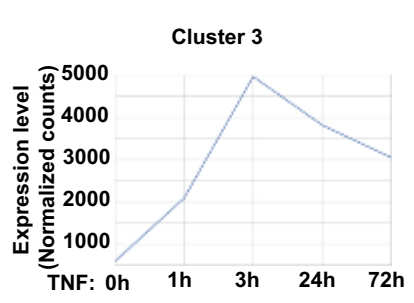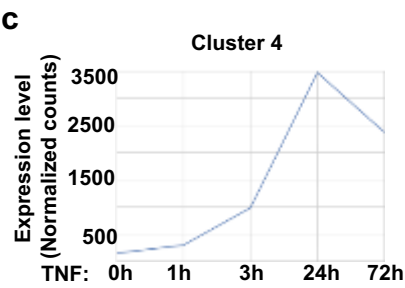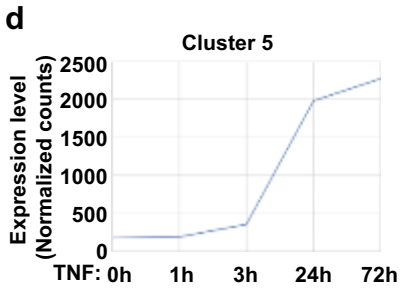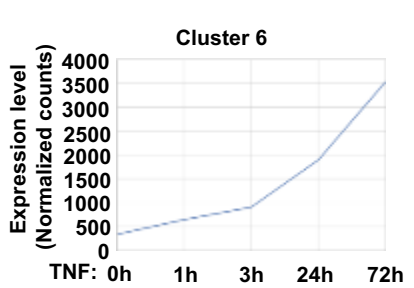

Supplement: S6 Fig — Two biological replicates of RA FLS (derived from two different RA patients) were exposed to TNF (10ng/ml) for 1-72h and gene expression was measured by RNA sequencing. 386 genes were identified as highly induced (≥5-fold) by TNF at any time point and were clustered into 6 clusters with distinct kinetics of peak expression. For (a-d), line graphs of mean expression (read counts) at 0-72h of TNF stimulation for each cluster. (PDF) [file pone.0179762.s007.pdf]
